# Assessing the performance of a *Fasciola gigantica* serum antibody ELISA to estimate prevalence in cattle in Cameroon

**DOI:** 10.1186/s12917-018-1762-z

**Published:** 2019-01-03

**Authors:** R. F. Kelly, S. Mazeri, C. Hartley, S. M. Hamman, V. Ngu Ngwa, E. F. Nkongho, V. Tanya, M. Sander, L. Ndip, K. L. Morgan, A. Muwonge, I. Handel, B. M. C. de Bronsvoort, D. J. L. Williams

**Affiliations:** 1 The Roslin Institute, Royal (Dick) School of Veterinary Studies, University of Edinburgh, Easter Bush, Midlothian, EH25 9RG UK; 2 Royal (Dick) School of Veterinary Studies, University of Edinburgh, Easter Bush, Midlothian, EH25 9RG UK; 3 Veterinary Parasitology, Institute of Infection and Global Health, Liverpool Science Park IC2, 146 Brownlow Hill, Liverpool, L3 5RF UK; 4 Institute of Agricultural Research for Development, Regional Centre of Wakwa, Ngaoundere, Cameroon; 5 School of Veterinary Medicine and Sciences, B.P. 454, University of Ngaoundere, Ngaoundere, Cameroon; 6 Microbiology and Parasitology Unit, Faculty of Allied Medical Science, University of Calabar, Calabar, Nigeria; 7 Cameroon Academy of Sciences, P.O. Box 1457, Yaoundé, Cameroon; 8 Tuberculosis Reference Laboratory Bamenda, Hospital Roundabout, Bamenda, Cameroon; 9 Laboratory of Emerging Infectious Diseases, University of Buea, Buea, Cameroon; 10 Institute of Ageing and Chronic Disease and School of Veterinary Science, University of Liverpool, Leahurst Campus, Neston, Wirral, CH64 7TE UK

**Keywords:** *Fasciola gigantica*, Fasciolosis, Cattle, Cameroon, Africa, Diagnostic test performance, Latent class methods, Bayesian non-gold standard methods

## Abstract

**Background:**

Cattle rearing in Cameroon is both economically and culturally important, however parasitic diseases detrimentally impact cattle productivity. In sub-Saharan Africa bovine fasciolosis is generally attributed to *F. gigantica*, although understanding of *Fasciola* species present and local epidemiology in individual countries is patchy. Partly limited by the lack of representative surveys and understanding of diagnostic test perfromance in local cattle populations. The aims of this paper were to determine the *Fasciola* species infecting cattle, develop a species specific serum antibody ELISA, assess the performance of the ELISA and use it to assess the prevalence of *F. gigantica* exposure in two important cattle-rearing areas of Cameroon.

**Results:**

A random sample of *Fasciola* parasites were collected and were all identified as *F. gigantica* (100%, CI:94.0–100%, *n* = 60) using RAPD-PCR analysis. A *F. gigantica* antibody ELISA was developed and initially a diagnostic cut-off was determined using a sample of known positive and negative cattle. The initial cut-off was used as starting point to estimate an optimal cut-off to estimate the best combination of sensitivity and specificity. This was achieved through sampling a naturally infected population with known infection status (cattle slaughtered at Bamenda abattoir, North West Region (*n* = 1112) and Ngaoundere abattoir, Vina Division, Adamawa Region (*n* = 776) in Cameroon). These cattle were tested and results analysed using a Bayesian non-gold standard method. The optimal cut-off was 23.5, which gave a sensitivity of 65.3% and a specificity of 65.2%. The prevalence of exposure to *F. gigantica* was higher in cattle in Ngaoundere (56.4% CI: 50.2–60.0%) than Bamenda (0.6% CI: 0.0–1.4%).

**Conclusion:**

*Fasciola gigantica* was identified as the predominant *Fasciola* species in Cameroon. Although the sensitivity and specificity *F. gigantica* antibody ELISA requires improvement, the test has shown to be a potentially useful tool in epidemiological studies. Highlighting the need for better understanding of the impact of *F. gigantica* infections on cattle production in Cameroon to improve cattle production in the pastoral systems of Central-West Africa. This paper also highlights that non-gold standard latent class methods are useful for assessing diagnostic test performance in naturally-infected animal populations in resource limited settings.

**Electronic supplementary material:**

The online version of this article (10.1186/s12917-018-1762-z) contains supplementary material, which is available to authorized users.

## Background

Infection with *Fasciola* species has been reported in a wide variety of mammalian species globally including cattle, sheep and humans [1]. Infection impacts on livestock production, animal health and welfare globally [2–5]. Chronic infections in cattle can result in disease, termed fasciolosis, presenting as weight loss, reduced milk yields and poor reproductive performance with an estimated global cost €2.5 million per annum [6]. Also bovine *Fasciola* species infections have recently been in the spotlight as bovine tuberculosis (bTB) and *Fasciola* species co-infected cattle have been shown to have down regulated Th1 immune responses affecting diagnosis of bTB [7, 8]. Frequent surveillance and forecasting of fasciolosis occurs in high income countries yet fasciolosis surveillance in many parts of sub-Saharan Africa (SSA) is minimal or infrequent [9–11]. Fasciolosis can be caused by two species of *Fasciola*, *F. hepatica* generally occurring in temperate or higher altitude tropical climates and *F. gigantica* limited to lower altitude tropical and sub-tropical climates [1, 12]. In most of SSA *F. gigantica* is believed to predominate, due to the ubiquitous presence of the intermediate hosts, including aquatic snail species such as *Lymnaea natalensis* [13–16].

Traditionally infection with *Fasciola* species is diagnosed either by faecal worm egg counts (FWEC), or by identification of parasites in the liver at post mortem examination (PME). FWECs are labour intensive and sensitivity can be low (69–90%) due to the volume of faeces produced by cattle and/or presence of immature parasites [1, 17]. Similarly, abattoir PME surveillance can miss early stages of *Fasciola* species [18]. Antibody diagnosis has been used to assess exposure to fluke in many countries and has the advantage of relatively high sensitivity (97–100%) and specificity (96–100%) [19] and ability to screen many cattle quickly. A variety of *Fasciola* antigens have been used to develop antibody diagnostics for use in ruminants based upon excretory/ secretory (E/S) antigens collected from adult *Fasciola* parasites [19–21]. However the majority of diagnostics have been developed for *F. hepatica* diagnosis with far fewer described specifically for *F. gigantica* [22–25].

There is a large population of approximately 6 million cattle in Cameroon which are integral economically, nutritionally and culturally to communities such as pastoralists predominately of Fulani origin, in the Northern Regions of Cameroon [26, 27]. These pastoral communities rear cattle in an extensive system on communal pasture and many still take their cattle on seasonal transhumance [28, 29]. Furthermore cattle trade is important not only nationally but to the wider central-west African region with cattle exports to neighbouring Nigeria, Gabon and Congo [30]. Previous reports suggest that *F. gigantica* occurs in cattle in Cameroon, but there have been few studies confirming that *F. gigantica* is the predominant species or that describe its local epidemiology [31–33]. Due to the potential economic impact on cattle production it is important to estimate levels of exposure and identify risk factors for *Fasciola* species infection. Improved understanding of the epidemiology of *F. giganica* may help identify interventions or particular risk regions where controls are most needed especially within these extensive pastoral grazing systems that extend to neighbouring countries. Hence our aims were to determine whether *F. gigantica* was the predominant species of *Fasciola* present in Cameroon and develop and assess the performance the *F. gigantica* antibody enzyme-linked immunosorbent assay (ELISA). Finally, to estimate the prevalence of *F. gigantica* in Cameroonian cattle populations.

## Results

### *Fasciola* species

**Fig. 1:**
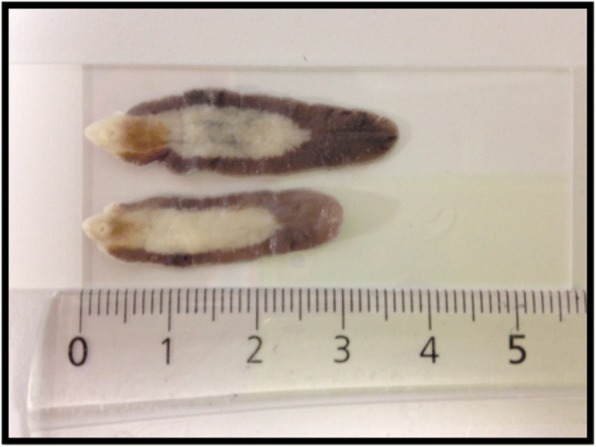
An image of two of the *Fasciola* parasites sampled (*n* = 60)

On gross appearance all parasites had characteristics of *F. gigantica* including slender shoulders and a long tapered shape (Fig. 1) [12, 34, 35]. Subsequently all *Fasciola* parasites tested by RAPD-derived sequence polymerase chain reaction (PCR) were identified as *F. gigantica* (100%, CI: 94.0–100%, *n* = 60).

### Development of a *Fasciola gigantica* antibody ELISA

All 20 parasites used to produce the two composite antigen preparations, ES101 and ES102, were identified as *F. gigantica*. Composite ES102 was selected to be used to develop the *F. gigantica* antibody ELISA (614 μg/ml) as the protein concentration of composite ES101 was low (100 μg/ml).

**Fig. 2:**
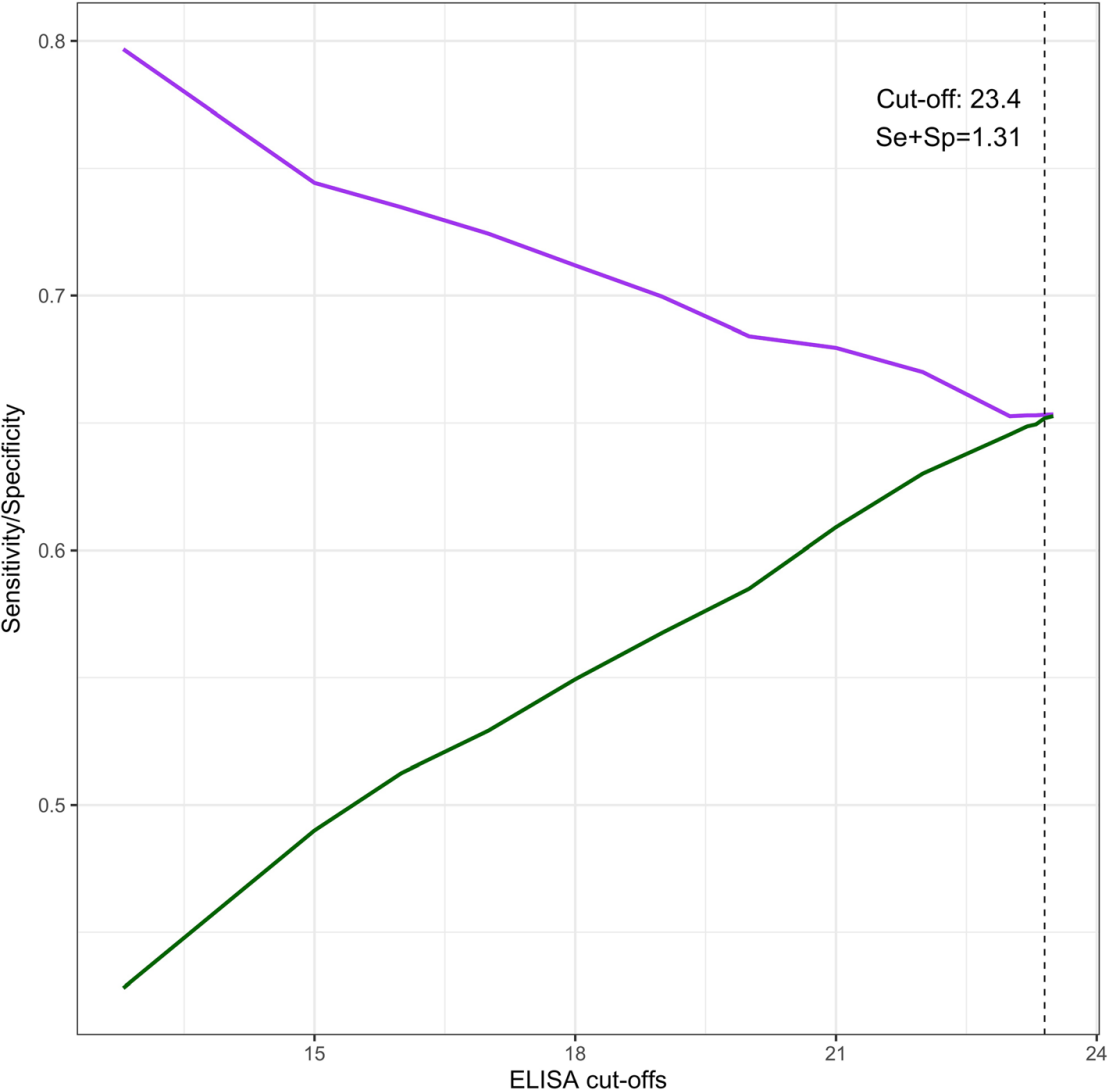
Sensitivity (Se) and specificity (Sp) estimates at various cut-offs using a Bayesian no gold standard analysis. Cut-offs used for analysis ranged from 12.8 PP to 23.5 PP. Sensitivity estimates are shown by the red line, while specificity estimates are shown by the green line. Cut-off 23.5 PP was chose as the final cut-off which gave the largest sum of Specificity and Sensitivity (1.31)

**Fig. 3:**
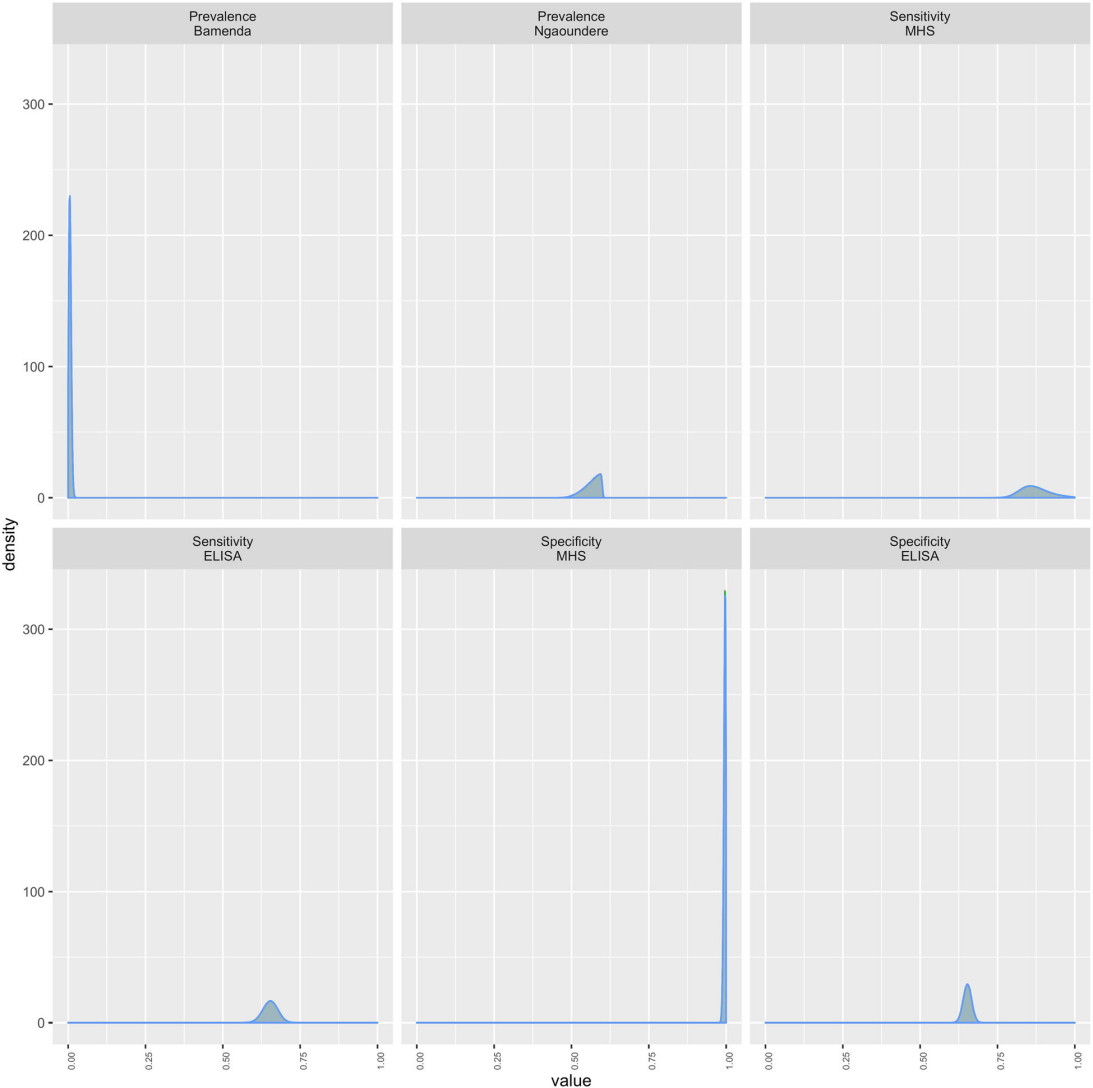
Posterior probability distributions of prevalence at Bamenda and Ngaounder abattoir and Sensitivity and Specificity estimates of the meat inspection (MHS) and *F. gigantica* ELISA estimated using Baysian no gold standard analysis

A total of 92 positive (*n* = 20) and negative (*n* = 72) cattle sera were tested using the ELISA. A receiver operating curve (ROC) was generated to select a positive cut-off value that achieved optimal sensitivity and specificity (Additional file 1). A positive cut-off value of 12.8% positive (PP) achieved best compromise in sensitivity (85.0%; 95% CI: 62.1–96.8%) and specificity (90.3%; 95% CI: 81.0–96.0%). This cut-off was then fine-tuned using a naturally infected cattle population from the abattoir cross-sectional study (*n* = 1888). A cut-off of 23.4 PP was chosen, which gave a sensitivity of 65.3% (95% CI 61.0–70.0%) and specificity of 65.2% (95% CI 62.5–67.8%). Figure 2 shows the range of cut-offs considered before the final cut-off was chosen and Fig. 3 shows the posterior probability distributions estimated by the NGS model. The posterior distributions represent the revised probability distribution of each parameter estimated by the model based on prior information and observed data.

### Prevalence of *Fasciola gigantica* in Cameroonian cattle

Subsequently, *F. gigantica* prevalence was estimated using no gold standard analysis using results from both meat inspection and the ELISA. *Fasciola gigantica* prevalence was higher in Ngaoundere abattoir, Vina Division (VD) (56.4% CI: 50.2–60.0%) than the Bamenda abattoir, North West Region (NWR) (0.6% CI: 0.0–1.4%).

## Discussion

This is the first time in Cameroon, and the Central-West African region, that *F. gigantica* infections in cattle have been confirmed using PCR techniques. The *F. gigantica* antibody ELISA had reasonable sensitivity and specificity. The abattoir cross sectional study showed that exposure to *F. gigantica* is common. All 60 parasites obtained from abattoirs in two different regions of Cameroon were identified as *F. gigantica*, suggesting that this species is the predominant *Fasciola* species in Cameroon. This confirms previous studies in the country where morphology was used to identify *F. gigantica* as the most prevalent species in the country [32, 33] and the surrounding Central-West African region [14, 15]. The distribution of *F. gigantica* is dictated by the distribution of the snail intermediate host, *L. natalensis*, which prefers humid tropical climates found across many regions of SSA [1, 36]. *L. truncatula*, the intermediate host of *F. hepatica* prefers cooler temperate climates. Geographical distribution of *Fasciola* species across Africa is only partially understood but pockets of *F. hepatica* do occur where the climate is favourable, especially in East Africa [36–39]. In Cameroon imports of European cattle, from East Africa and outside the continent, could have brought *F. hepatica* into areas where the intermediate host is present [40, 41]. However, our abattoir study shows no evidence of *F. hepatica* in four major cattle rearing Regions in Cameroon.

The *F. gigantica* ELISA had a diagnostic sensitivity of 65.3% (95% CI 61.0–70.0%) and specificity of 65.3 65.2% (95% CI 62.5–67.8%) at a cut off of 23.4 PP. The sensitivity and specificity for our test were lower than other *F. gigantica* antibody detection ELISAs (Sensitivity: 81.8–100% and specificity: 91.6–98.9% [23, 42–45]) and the *F. hepatica* ELISA it was based on (sensitivity: 98.0% CI: 96.0–100% and specificity: 98.90% CI: 93.0–98.0% [24]). To assess the diagnostic performance of our ELISA, we used a large sample (*n* = 1888) of natural infected cattle with information on liver rejections due to *Fasciola* infection by meat inspectors. This enabled us to use a non-gold standard diagnostic test comparison approach. This is the first time a non-gold standard approach has been used for assessessing *Fasciola* diagnostics in this environment and has the benefit of assessing the diagnostic performance in a real-world setting, where a variety of disease states and parasitic burdens exist. Although there are limitations to this approach, this method provides a more realistic estimate of the performance of this diagnostic test for estimating prevalence and highlights areas for further improvement.

The lower estimated sensitivity and specificity could be influenced by factors associated with the sample of animals tested. For the animals that were defined negative by the ELISA, it is possible that liver damage was caused by other infectious agents or that the *F. gigantica* burden was very low. The magnitude of the antibody responses has been shown to correlate with burden of *F. gigantica* in cattle [46] and cattle infected with low burdens of *F. hepatica* have been shown to have absent or lower E/S antigen specific Immunoglobulin G (IgG) responses [19]. Here the *F. gigantica* burden of positive cattle was unknown and is likely to have fluctuated throughout their lives. IgG responses may be influenced by fluctuations in total parasite burden overtime [47]. Cross-reactions with other helminth infections could have contributed to false positives. Rumen flukes, such as *Calicophoron daubneyi* and *Paramphistomum cervi,* have been reported to cross-react with *F. hepatica* in ELISAs [22, 48]. In our study, cattle sampled in the abattoir cross-sectional study were not tested for other co-infections. Hence cross-reactions with other parasites, particularly other trematodes such as schistosomes and rumen flukes present in Cameroon, could not be ruled out in this study. It is also possible that cattle that have been treated with anthelmintic can have a residual antibody responses despite effective anthelmintic treatment [46, 49]. Albendazole, which is effective against adult *Fasciola* parasites, is available in Cameroon and it was unknown if cattle in the abattoir study were treated with anthelmintic. It is possible that cattle defined as *Fasciola* negative on post-mortem, could have had previous *Fasciola* infection at low enough levels to stimulate a historic antibody response. Thus, our results highlight that test performance of *F. gigantica* ELISAs should be further investigated across the variety of infection and disease states, to avoid over-estimation of test performance in naturally infected populations.

By using a non-gold standard approach, we can take into account real-world test performance to estimate prevalence of exposure to *F. gigantica* in cattle in Cameroon. Using this latent class method, a very prevalence was found in the Ngaoundere abattoir, VD (56.4% CI: 50.2–60.0%) and to a lesser extent in Bamenda abattoir, NWR (0.6% CI: 0.0–1.4%). Bovine *F. gigantica* infections have been associated with 22 kg per animal reduction in live-weight gains in yearling cattle [50] and economic losses due to liver condemnations [51]. Poor weight gain has been highlighted as a production-limiting factor in cattle production in Cameroon [52] and could be partly attributed to active *F. gigantica* infections. Previous abattoir studies have reported higher prevalence in slaughtered cattle in Cameroon (~ 80%) [32, 33, 53]. Variability in prevalence could be related seasonaility of *F. gigantica* infections, time of year cattle are sampled and which cattle are sold for slaughter. For example, when cattle are sold for slaughter at markets in Cameroon, cattle are often sold for slaughter by pastoralists due to ill health and poor weight [54] which could be related to a variety of diseases other than fasciolosis. As previously mentioned, the majority of cattle slaughtered in Bamenda abattoir originated from the NWR and those slaughtered in Ngaoundere abattoir from the VD. The results of this study suggest that the VD had a higher seroprevalence than the NWR. Foci of higher prevalence could be related to local climatic conditions for intermediate host stages and local cattle husbandry practices [55, 56]. Improved performance of the *F. gigantica* ELISA would be useful to identify risk factors for exposure to identify infection control points in Cameroon. Future work should investigate the impact of parasite burden/ stage post-exposure on ELISA sensitivity and co-infection cross reactions that may affect ELISA specificity.

## Conclusions

Although the sensitivity and specificity *F. gigantica* antibody ELISA need improvement, the test has shown to be a potentially useful tool in epidemiological studies where *F. gigantica* exposure is a concern in cattle populations. *Fasciola gigantica* was identified as the predominant *Fasciola* species in Cameroon and a seroprevalence study showed that over a third of cattle show evidence of exposure. Whilst herdsmen are aware of fasciolosis, the economic and health costs to cattle production remain unclear [28]. With increasing demand for animal protein in the country [57], understanding the impact of *F. gigantica* infections on cattle production in Cameroon is needed to determine if better control strategies are required to improve cattle production in the pastoral systems of Central-West Africa.

## Methods

### Determination of *Fasciola* species by RAPD-derived sequence PCR

**Fig. 4:**
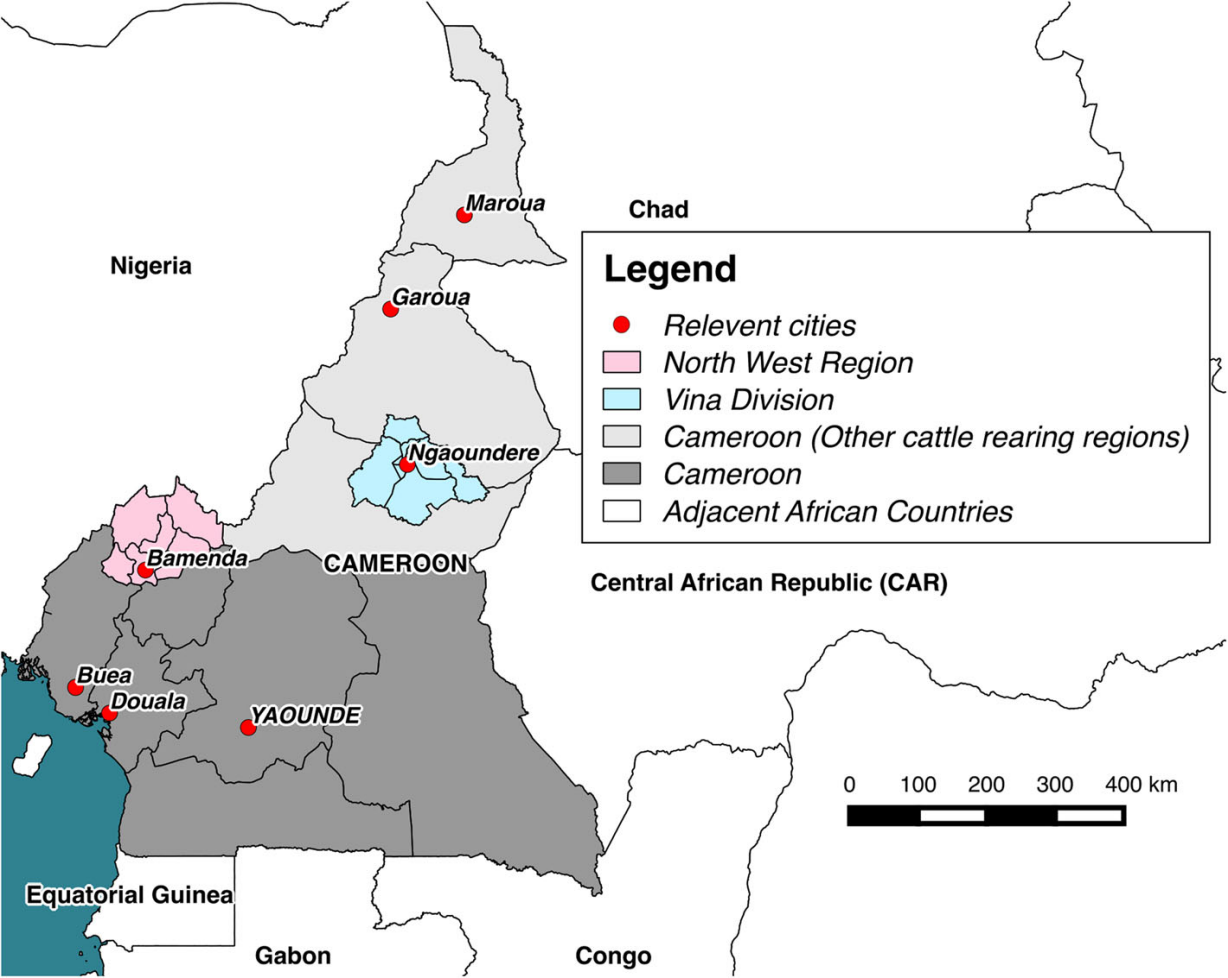
A map of Cameroon (grey) with the main cattle rearing areas highlighted (light grey) and adjacent countries (white). The location of four regional abattoirs, the country’s capital (italic capital letters) and other major cities are highlighted (red). The North West Region (NWR) and Vina Division (VD) study sites are shown in pink and blue respectively

A convenience sample of *Fasciola* species parasites were collected from cattle from four abattoirs in Cameroon during 2012–2013 (Fig. 4). Abattoirs included Bamenda central municipal abattoir, North West Region (NWR) in February–July 2012 (*n* = 35; including 20 from which E/S antigen was collected), Ngaoundere central municipal abattoir, Vina Division (VD), Adamawa Region (AR) in August 2013 (*n* = 15), Garoua central municipal abattoir, North Region in October 2013 (*n* = 5) and Maroua central municipal abattoir, Extreme North Region in November 2013 (n = 5). The basic physical appearance of whole parasites was noted and compared to gross descriptions of *F. hepatica* and *F. gigantica* parasites [12, 34, 35]. Live adult *Fasciola* parasites were washed six times in sterile PBS to remove liver tissue, bile and blood. Parasites were then preserved in 70–100% ethanol at time of collection, stored at -20 °C in the Tuberculosis Reference Laboratory (TBRL), Bamenda, Cameroon. Samples were subsequently transported to the Veterinary Parasitology Department, University of Liverpool, Liverpool UK to determine *Fasciola* species.

Determination of *Fasciola* species was conducted using two RAPD-derived sequence PCRs for *F. gigantica* and *F. hepatica* (*n* = 60) using the method described by McGarry et al. (2007). Briefly, 25 μg of tissue was removed from the caudal portion of each parasite. Each sample was washed six times in sterile PBS and ethanol was allowed to evaporate for two hours before genomic DNA was extracted using a DNeasy® blood and tissue kit (QIAGEN® Crawley, UK) and used the same day. Primer set one for *F. hepatica*, was Forward primer: 5’GCG GCC AAA TAT GAG TCA-3′ and reverse primer: 5’-CTG GAG ATT CCG GTT ACC AA-3′ of 568 bp. Primer set two, for *F. gigantica*, Forward primer: 5′-GTT CAG GTG ACA AGC CAA-3′ and reverse primer: 5’-ATC ACA CCG TGA AGC AGA-3′ of 396-bp. Each PCR contained 12.5 μl of BioMix®Red (1X, Bioline®, Sydney, Australia), 0.5 μl of 10 μmol forward primer from set one or two, 0.5 μl of 10 μmol reverse primer set one or two (final concentration of both primers 0.2 μmol, 10.5 μl of sterile water and 1.0 μl of template DNA (> 10 ng) from sampled parasites (Total volume 25 μl). For the *F. hepatica* primer set, the thermocycler (Biometra® T3 Thermocycler) PCR conditions were 95 °C initial denaturation for 15mins; 30 cycles of 94 °C for 30s, 54 °C for 40s and 72 °C for 1 min; and a final 72 °C 3 min extension. For the *F. gigantica* primer set PCR, the thermocycler was set at 95 °C for 15mins; 30 cycles of 94 °C for 30s, 56 °C for 40s and 72 °C for 1 min; a final 72 °C 3 min extension. The positive *F. hepatica* control was from a cow in the UK infected with *F. hepatica*. Positive *F. gigantica* control was from a Ugandan cow naturally infected with *F. gigantica*. A sample of bovine DNA and a sample of sterile water were used as negative controls in each PCR and a 100 bp DNA ladder (GENEFLOW®) was used. PCR products were stained with 10 μl in 100 ml SYBRsafe® (1X, Life technologies®) and separated in 1.5% agarose gel for 45mins at 150 V. Separated PCR products were visualised in ultraviolet light (UV) by trans-illumination. PCR results were compared to determine the species of parasite.

### Development of a *Fasciola gigantica* serum antibody ELISA

#### E/S antigen collection

A total of 92 live *Fasciola* species parasites were collected from two cattle [34, 48] slaughtered at Bamenda abattoir and these parasites were used to produce the E/S antigen. Antigen preparation was undertaken in the TBRL, Bamenda, NWR, Cameroon. Live parasites were washed six times in sterile PBS and then six times with RPMI tissue culture medium to remove liver tissue, bile and blood with being allowed to purge for an hour prior incubation. Parasites were then incubated in 1 ml of RPMI medium for 12 h at 37 °C in a humidified atmosphere in a poultry hatching incubator (Hova-Bator® Incubator 37C&60E). At the end of the incubation, the flukes were checked to confirm they were still alive and then the supernatant was collected and centrifuged at 10,000 g for 30 min at 4 °C to remove particulate material. Supernatants were further filtered by passing through individual 0.22 μm filters (Sartorius, Minisart® 16,532 K). Samples were stored at -20 °C in aliquots and transported refrigerated to the Roslin Institute, Royal (Dick) School of Veterinary Studies (RDSVS), University of Edinburgh, UK. The parasites from each well were stored in 100% ethanol and the RAPD PCR used to establish the species of each individual parasite.

E/S antigen supernatants from 10 *F. gigantica,* confirmed by PCR, were pooled into two composites. Protein concentration of E/S antigen composites was measured using a total protein assay (Coomassie Plus (Bradford) assay, Thermo Scientific®). A protein concentration of 100-1500μg/ml was considered adequate for use in the ELISA protocol.

#### ELISA laboratory method

A protocol used for a *F. hepatica* antibody ELISA was modified to develop a *F. gigantica* antibody ELISA using E/S antigen as described [24]. Immulon-2 ELISA 96-well plates were coated with 100 μl of 1 μg/ml *F. gigantica* E/S antigen in 0.1 M carbonate buffer (pH 9.6). Plates were incubated for 1 h at room temperature and then refrigerated at 2-4 °C overnight. Plates were then washed six times (Two short washes and one five minute wash repeated twice) with pH 7.2 PBS containing 0.05% Tween-20 (PBS-Tween). Each well was then blocked with 200ul of blocking buffer for 1 h at 37 °C (4% skimmed powder (Marvel, Premier International Foods®, Spalding UK) in PBS-Tween. Plates were washed six times, and 100 μl of sera diluted 1:200 in blocking buffer added to each well. The positive control used was serum collected from an animal identified in Bamenda abattoir positive for *F. gigantica* infection at meat inspection and confirmed as *F. gigantica* by PCR. A *Fasciola* species negative cow, which had been kept indoors throughout her life at Ness Heath Farm (University of Liverpool, Cheshire UK), was used as the negative control serum. Positive and negative controls were added to the plate in duplicate, at the same concentration as the test sera, and incubated at 37 °C for 1 h. The plates were again washed and 100 μl of 1:1500 mouse anti-bovine IgG HRP conjugate (Serotec®, UK) in blocking buffer added then the plates were incubated at 37 °C for 1 h. After washing, 100 μl of TMB substrate (Acetate buffer pH 5 and tetramethylbenzidine in a methanol based solution, MAST Diagnostics, Bootle, Merseyside, UK) was added and incubated at room temperature for 20 mins in the dark. Finally 100 μl of stopping solution (10% Hydrochloric acid) was added and the colour change measured at 450 nm using an automated microplate reader (Thermoscientific® Multiskan Go). The results were obtained as an optical density (OD) and expressed as a PP value:

$$ \mathrm{PP}=\frac{\mathrm{OD}\;\mathrm{of}\kern0.17em \mathrm{test}\kern0.17em \mathrm{sample}}{\mathrm{Mean}\;\mathrm{OD}\;\mathrm{of}\kern0.17em \mathrm{positive}\kern0.17em \mathrm{control}}\mathrm{X}\;100 $$

#### Known positive and negative cattle

To calculate an initial diagnostic cut-off value for the ELISA, serum samples from cattle of known positive and negative *F. gigantica* status were used. Known naturally infected *F. gigantica* positive cattle (*n* = 20) were identified at meat inspection and cases were confirmed grossly by investigators (R. F. Kelly and S. Mazeri) as part of this study. Ten cattle from Bamenda abattoir and 10 cattle from Ngaoundere abattoir were used in the analysis. The *F. gigantica* negative cattle (*n* = 72) included cattle sampled in the United Kingdom (UK) and Cameroon. Due to differences in sampling methodology, the definition of known negative status differed for cattle sampled in the UK and Cameroon. Cattle sampled in the UK were kindly donated from a cross-sectional abattoir study conducted in 2013–14 (*n* = 20) [48]. Cattle were known to be *Fasciola* species negative by FWEC, detailed liver PME, *F. hepatica* serum antibody ELISA and *F. hepatica* copro-antigen ELISA [19, 24]. Furthermore *F. gigantica* has never been diagnosed in UK cattle populations. To increase the sample size an additional 57 dairy cattle sampled in Cameroon, which had all been treated with anthelmintic (Albendazole) in the past 12 months and kept housed during that time, were assumed for the purposes of this analysis to be *F. gigantica* negative.

#### Abattoir cross-sectional study

To determine the optimal diagnostic cut off value for the ELISA, the test was further evaluated and prevalence estimated using samples from a naturally infected cattle population. Slaughtered cattle were sampled from Bamenda and Ngaoundere abattoirs during a cross-sectional study investigating bovine tuberculosis [58]. This included 1112 cattle from Bamenda abattoir and 776 from Ngaoundere abattoirs, originating from the local area [58]. As previously described, cattle slaughtered in Bamenda and Ngaoundere abattoirs originated from the NWR and part of the AR respectly. For all slaughted cattle, liver inspection by the meat hygiene service for evidence of *Fasciola* infection was recorded and whole blood samples were taken using 6 ml plain vacutainers. Whole blood samples were stored between 10 °C to 26 °C for up to 24 h to allow blood to clot. The vacutainers were centrifuged at 3000 g for 10 min at room temperature (22 °C +/-5 °C) to separate the serum. After centrifugation 0.5–1.5 ml of serum per vacutainer was transferred into a pre-labelled 1.8 ml cryovial and stored at -20 °C. Serum samples were heat treated at 56 °C in a waterbath. Serum samples were then transported to the UK, continued to be stored at -20 °C, and at the Roslin Institute (RDSVS, Edinburgh, UK) and subsequently tested using the ELISA in March–April 2014.

### Statistical analysis

Statistical analyses were performed using several packages of the R Statistical Software environment [59]. Graphics were produced using the ggplot2 [60] and ggmcmc [61] packages.

#### Determining a positive cut off value

Samples of known positive or negative status as described above were used to identify an initial cut-off for the ELISA using a ROC analysis. This analysis selects a suitable positive cut-off OD value based on the best combination of sensitivity and specificity of the *F. gigantica* ELISA. ROC curve was plotted using the *pROC* package and was used to estimate the optimal cut-off point for the ELISA [62].

#### No gold standard diagnostic test comparison

A Bayesian no gold standard (NGS) approach was used to estimate the diagnostic ability of *F. gigantica* ELISA in a naturally infected cattle population using binary results of meat inspection and ELISA on cattle sampled in the two abattoirs. This approach, introduced by Hui & Walter [63], is a latent class approach that enables the evaluation of diagnostic tests when a “gold standard” is not available. Probabilities of all the possible combinations of test outcomes conditional on the unknown disease status are specified using the sensitivity (Se) and specificity (Sp) of each test (1 - Meat inspection and 2 - ELISA) and the prevalence (p) of each sub-population (Bamenda and Ngaoundere abattoir) [48]. For example, the probability of obtaining a positive result by meat inspection and negative by the ELISA would be:

$$ \Pr \left(\mathrm{T}1+,\mathrm{T}2-\right)=\mathrm{Se}{1}^{\ast }\ {\left(1-\mathrm{Se}2\right)}^{\ast }\ \mathrm{p}+{\left(1-\mathrm{Sp}1\right)}^{\ast }\ \mathrm{Sp}2\ \left(1-\mathrm{p}\right) $$

The Bayesian adaptation incorporates prior knowledge by specifying prior distributions for test sensitivities, specificities and prevalence. For this model, we used vague priors for all estimates except the specificity of meat inspection which is known to be very high [48]. Priors used are shown in Table 1. Starting from the cut-off identified using a limited number of known negative and positive samples, the no gold standard analysis was repeated using a range of cut-offs until the optimal Se and Sp were identified. This model also enabled us to estimate prevalence of exposure to *Fasciola* in animals slaughtered in the two abattoirs.

**Table 1:** Priors used for No Gold Standard analysis

Estimates	Prior
prevalence	dunif(0, 0.6)
Se (meat inspection)	dbeta [1]
Sp (meat inspection)	dbeta [1, 9]
Se (ELISA)	dbeta [1]
Sp (ELISA)	dbeta [1]

## Additional file

Additional file 1: The receiver operator curve (ROC) for the *F. gigantica* antibody ELISA of *F. gigantica* positive (*n* = 20) and negative (*n* = 72) cattle. The SE and SP of the ELISA is represented on the y and x axes respectively. The line (“Staircase trace”) represents SE and SP, with 95% CI being the grey shaded area, at different positive cut-off values for the ELISA. A selected positive cut-off value of 12.8 PP balances sensitivity (85.0%) and specificity (90.3%) with an AUC of 83.6%. (TIFF 42 kb)

## Abbreviations

AR: Adamawa Region
E/S: Excretory/ secretory
ELISA: Enzyme-linked immunosorbent assay
FWEC: Faecal worm egg count
IgG: Immunoglobulin G
IRAD: Institute of Research and Development
NGS: Non-gold standard
NWR: North West Region
OD: Optical density
PCR: Polymerase chain reaction
PME: Post mortem examination
PP: Percent positive
ROC: Receiver operating curve
Se: Sensitivity
Sp: Specificity
SSA: Sub-Saharan Africa
TBRL: Tuberculosis Reference Laboratory
UK: United Kingdom

## References

[1] Torgerson P, Claxton J, Dalton JP (1999). Epidemiology and control. Fasciolosis.

[2] Legesse G, Asfaw Y, Tolossa Y, Beyene T. Epidemiology and economic importance of fasciolosis of domestic ruminants in selected districts of Tigray Region, Ethiopia [Internet]. Vol. 18, Ethiopian Veterinary Journal. Ethiopian Veterinary Association; 2015 [cited 2015 Jul 23]. p. 51–64. Available from: http://www.ajol.info/index.php/evj/article/view/119525

[3] Khan MK, Sajid MS, Riaz H, Ahmad NE, He L, Shahzad M, et al. The global burden of fasciolosis in domestic animals with an outlook on the contribution of new approaches for diagnosis and control. Parasitol Res [Internet]. 2013 Jul [cited 2015 Jul 23];112(7):2421–2430. Available from: https://link.springer.com/article/10.1007%2Fs00436-013-3464-6 10.1007/s00436-013-3464-6 23728732

[4] Khaniki GRJ, Kia EB, Raei M. Liver condemnation and economic losses due to parasitic infections in slaughtered animals in Iran. J Parasit Dis [Internet]. [cited 2015 23];37(2):240–4. Available from: https://www.ncbi.nlm.nih.gov/pmc/articles/PMC3793095/pdf/12639_2012_Article_172.pdf. Accessed 28 Dec 2018. 10.1007/s12639-012-0172-6 PMC3793095 24431577

[5] Bennett RM, Christiansen K, Clifton-Hadley RS. Modelling the impact of livestock disease on production: case studies of non-notifiable diseases of farm animals in Great Britain. Anim Sci [Internet]. 1998 [cited 2015 Jul 23];68(4):681–9. Available from: https://www.cambridge.org/core/journals/animal-science/article/modelling-the-impact-of-livestock-disease-on-production-case-studies-of-nonnotifiable-diseases-of-farm-animals-in-great-britain/F7908A3252DBB20B087D20E46073BE56

[6] Innovation. D-G for R and. A decade of EU-funded Animal Health. Luxembourg; 2012.

[7] Flynn RJ, Mulcahy G, Welsh M, Cassidy JP, Corbett D, Milligan C, et al. Co-Infection of cattle with Fasciola hepatica and Mycobacterium bovis- immunological consequences. Transbound Emerg Dis [Internet]. 2009 [cited 2011 Sep 27];56(6–7):269–74. Available from: https://onlinelibrary.wiley.com/doi/full/10.1111/j.1865-1682.2009.01075.x. 10.1111/j.1865-1682.2009.01075.x 19575746

[8] Kelly RF, Callaby R, Egbe NF, Williams DJL, Victor NN, Tanya VN, et al. Association of *Fasciola gigantica* Co-infection With Bovine Tuberculosis Infection and Diagnosis in a Naturally Infected Cattle Population in Africa. Front Vet Sci [Internet]. 2018 6 [cited 2018 Sep 6];5:214. Available from: https://www.frontiersin.org/article/10.3389/fvets.2018.00214/full 10.3389/fvets.2018.00214 PMC6136300 30238010

[9] McCann CM, Baylis M, Williams DJL. The development of linear regression models using environmental variables to explain the spatial distribution of *Fasciola hepatica* infection in dairy herds in England and Wales. Int J Parasitol [Internet]. 2010 1 [cited 2015 Jul 23];40(9):1021–8. Available from: https://www.sciencedirect.com/science/article/pii/S0020751910000676?via%3Dihub 10.1016/j.ijpara.2010.02.009 20227416

[10] Goodall EA, McIlroy SG, McCracken RM, McLoughlin EM, Taylor SM. A mathematical forecasting model for the annual prevalence of Fasciolosis. Agric Syst [Internet]. 1991 Jan [cited 2015 Jul 23];36(2):231–40. Available from: http://www.sciencedirect.com/science/article/pii/0308521X91900267

[11] Yilma JM, Malone JB. A geographic information system forecast model for strategic control of fasciolosis in Ethiopia. Vet Parasitol [Internet]. 1998 31;78(2):103–27. Available from: https://www.sciencedirect.com/science/article/pii/S0304401798001368?via%3Dihub. 10.1016/s0304-4017(98)00136-8 9735916

[12] Taylor MA, Coop RL, Wall RL (2007). Veterinary parasitology. 3rd Editio.

[13] Abunna F, Asfaw L, Megersa B, Regassa A. Bovine fasciolosis: coprological, abattoir survey and its economic impact due to liver condemnation at Soddo municipal abattoir, Southern Ethiopia. Trop Anim Health Prod [Internet]. 2010 [cited 2011 Dec 13];42(2):289–92. Available from: https://link.springer.com/article/10.1007%2Fs11250-009-9419-3. 10.1007/s11250-009-9419-3 19680772

[14] Opara KN (2005). Population dynamics of Fasciola gigantica in cattle slaughtered in Uyo. Nigeria Parasitology.

[15] Tembeley S, Galvin TJ, Craig TM, Traore SI (1988). Liver fluke infections of cattle in Mali. An abattoir survey of prevalence and geographic distribution. Trop Anim Health Prod.

[16] Hammond RF. Infections with Fasciola spp. in wildlife in Africa. Trop Anim Health Prod [Internet]. 1972 [cited 2015 23];4:1–13. 10.1007/BF02357089 4671397

[17] Anderson N, Luong TT, Vo NG, Bui KL, Smooker PM, Spithill TW. The sensitivity and specificity of two methods for detecting Fasciola infections in cattle. Vet Parasitol [Internet]. 1999 Jun [cited 2015 Jul 5];83(1):15–24. Available from: http://www.sciencedirect.com/science/article/pii/S0304401799000266 10.1016/s0304-4017(99)00026-6 10392764

[18] Bennema SC, Ducheyne E, Vercruysse J, Claerebout E, Hendrickx G, Charlier J. Relative importance of management, meteorological and environmental factors in the spatial distribution of *Fasciola hepatica* in dairy cattle in a temperate climate zone. Int J Parasitol [Internet]. 2011 Feb [cited 2015 Jun 11];41(2):225–33. Available from: http://www.sciencedirect.com/science/article/pii/S0020751910003164 10.1016/j.ijpara.2010.09.003 20887726

[19] Charlier J, De Meulemeester L, Claerebout E, Williams D, Vercruysse J. Qualitative and quantitative evaluation of coprological and serological techniques for the diagnosis of fasciolosis in cattle. Vet Parasitol [Internet]. 2008 [cited 2011 Sep 26];153(1–2):44–51. Available from: https://www.sciencedirect.com/science/article/pii/S030440170800071X?via%3Dihub. 10.1016/j.vetpar.2008.01.035 18329811

[20] Hillyer GV, Dalton JP (1999). Immunodiagnosis of human and animal fasciolosis.

[21] Leclipteux T, Torgerson PR, Doherty ML, McCole D, Protz M, Farnir F, et al. Use of excretory/secretory antigens in a competition test to follow the kinetics of infection by *Fasciola hepatica* in cattle. Vet Parasitol [Internet]. 1998 15;77(2–3):103–14. Available from: https://www.sciencedirect.com/science/article/pii/S0304401798001034?via%3Dihub. 10.1016/s0304-4017(98)00103-4 9746280

[22] Ibarra F, Montenegro N, Vera Y, Boulard C, Quiroz H, Flores J, et al. Comparison of three ELISA tests for seroepidemiology of bovine fascioliosis. Vet Parasitol [Internet]. 1998 Jun [cited 2015 Jul 23];77(4):229–36. Available from: https://www.sciencedirect.com/science/article/pii/S0304401798001113?via%3Dihub 10.1016/s0304-4017(98)00111-3 9763313

[23] Molloy JB, Anderson GR, Fletcher TI, Landmann J, Knight BC. Evaluation of a commercially available enzyme-linked immunosorbent assay for detecting antibodies to Fasciola hepatica and *Fasciola gigantica* in cattle, sheep and buffaloes in Australia. Vet Parasitol [Internet]. 2005 [cited 2014 Feb 12];130(3–4):207–12. Available from: https://www.sciencedirect.com/science/article/pii/S0304401705001287?via%3Dihub. 10.1016/j.vetpar.2005.02.010 15925723

[24] Salimi-Bejestani MR, McGarry JW, Felstead S, Ortiz P, Akca A, Williams DJL. Development of an antibody-detection ELISA for Fasciola hepatica and its evaluation against a commercially available test. Res Vet Sci [Internet]. 2005 [cited 2012 Jan 12];78(2):177–81. Available from: https://www.sciencedirect.com/science/article/pii/S0034528804001559. 10.1016/j.rvsc.2004.08.005 15563926

[25] Youssef FG, Mansour NS (1991). A purified Fasciola fascioliasis gigantica worm antigen for the serodiagnosis of human. Trans R Soc Trop Med Hyg.

[26] Burnham P. The Mbororo ethic catagory: distinction or exclusion? In: Peel JDY, Parkin D, editors. The politics of cultural difference in northern Cameroon. First Edit. Edinburgh University Press; 1996. p. 96–116.

[27] Eyongetah T, Brian R. 5. The peopling of Cameroon. In: The History of Cameroon. First. Boston, MA, USA: Longman; 1974. p. 19–26.

[28] Kelly RF, Hamman SM, Morgan KL, Nkongho EF, Ngwa VN, Tanya V, et al. Knowledge of Bovine Tuberculosis, Cattle Husbandry and Dairy Practices amongst Pastoralists and Small-Scale Dairy Farmers in Cameroon. Chang Y-F, editor. PLoS One [Internet]. 2016;11(1):e0146538. Available from: http://journals.plos.org/plosone/article?id=10.1371/journal.pone.0146538 10.1371/journal.pone.0146538 PMC4706344 26745871

[29] Bronsvoort BM deC, Tanya VN, Kitching RP, Nfon C, Hamman SM, Morgan KL. Foot and mouth disease and livestock husbandry practices in the Adamawa Province of Cameroon. Trop Anim Health Prod [Internet]. 2003 Dec;35(6):491–507. Available from: https://link.springer.com/article/10.1023/A:1027302525301. 10.1023/a:1027302525301 14690088

[30] United Nations Statistics Division. UN data: Cameroon [internet]. 2014. Available from: http://data.un.org/en/iso/cm.html

[31] Ntonifor HN, Shei SJ, Ndaleh NW, Mbunkur GN. Epidemiological studies of gastrointestinal parasitic infections in ruminants in Jakiri, Bui Division, North West Region of Cameroon. J Vet Med Anim Heal [Internet]. [cited 2015 Jul 23];5(12):344–52. Available from: http://citeseerx.ist.psu.edu/viewdoc/download?doi=10.1.1.428.8247&rep=rep1&type=pdf. Accessed 28 Dec 2018.

[32] Ntonifor HN, Ndaleh WN. Prevalence of liver fluke infections and other gastrointestinal tract parasites in slaughtered cattle in Douala, Cameroon. Bull Anim Heal Prod Africa [Internet]. [cited 2015 Jul 23];60(4):438–44. Available from: https://www.ajol.info/index.php/bahpa/article/view/91848. Accessed 28 Dec 2018.

[33] Ngole IU, Ndamukong KJN, Mbuh J V. Short Communication Internal Parasites and Haematological Values in Cattle Slaughtered in Buea Subdivision of Cameroon. Heal (San Fr. 2003;35:409–13. 10.1023/a:1025811428008 14620585

[34] Shaba GH, Arfaa F, Farahmandian I, Jalali H (1972). Animal fasciolosis in Khuzestan. Southwestern Iran J Parasitol.

[35] Lotfy WM, El-Morshedy HN, Abou El-Hoda M, El-Tawila MM, Omar EA, Farag HF (2002). Identification of the Egyptian species of Fasciola. Vet Parasitol.

[36] Hubendick B. Recent Lymnaeidae. Their variation, morphology, taxonomy, nomenclature and distribution. K Sven VETENSKAPSAKAD HANDL [Internet]. 1951 [cited 2015 Jul 24];3(1):1–225.

[37] Walker SM, Makundi AE, Namuba FV, Kassuku AA, Keyyu J, Hoey EM, et al. The distribution of Fasciola hepatica and *Fasciola gigantica* within southern Tanzania--constraints associated with the intermediate host. Parasitology [Internet]. 2008 [cited 2011 Dec 13];135(4):495–503. Available from: https://www.cambridge.org/core/journals/parasitology/article/distribution-of-fasciola-hepatica-and-fasciola-gigantica-within-southern-tanzania-constraints-associated-with-the-intermediate-host/19CF677228A64ED82F041BA6E953797F. 10.1017/S0031182007004076 18205983

[38] Ali H, Ai L, Song HQ, Ali S, Lin RQ, Seyni B, et al. Genetic characterisation of Fasciola samples from different host species and geographical localities revealed the existence of F. hepatica and *F. gigantica* in Niger. Parasitol Res [Internet]. 2008 [cited 2011 Oct 17];102(5):1021–4. Available from: https://link.springer.com/article/10.1007%2Fs00436-007-0870-7. 10.1007/s00436-007-0870-7 18183422

[39] Howell A, Mugisha L, Davies J, LaCourse EJ, Claridge J, Williams DJL, et al. Bovine fasciolosis at increasing altitudes: parasitological and malacological sampling on the slopes of Mount Elgon, Uganda. Parasit Vectors [Internet]. 2012 Jan [cited 2014 Feb 12];5(1):196. Available from: https://parasitesandvectors.biomedcentral.com/articles/10.1186/1756-3305-5-196 10.1186/1756-3305-5-196 PMC3464174 22958402

[40] Awah-Ndukum J, Kudi AC, Bradley G, Smith NH, Ane-Anyangwe I, Cho-Ngwa F, et al. Molecular genotyping of Mycobacterium bovis isolated from cattle tissues in the North West Region of Cameroon. Trop Anim Health Prod [Internet]. 2012 Nov 7 [cited 2012 Nov 10]; Available from: https://link.springer.com/article/10.1007%2Fs11250-012-0295-x. 10.1007/s11250-012-0295-x 23132133

[41] Njew RM, Kwinji LN, Gabache AL, Tambi EN. Contributions of Heifer Project International (HPI) to small-scale dairy development in Cameroon. In: Smallholder dairy production and marketing- opportunities and constraints [Internet]. Nairobi; 2002. Available from: https://cgspace.cgiar.org/bitstream/handle/10568/16607/SS_Proceeding.pdf?sequence=1

[42] Awad WS, Ibrahim AK, Salib FA. Using indirect ELISA to assess different antigens for the serodiagnosis of *Fasciola gigantica* infection in cattle, sheep and donkeys. Res Vet Sci [Internet]. 2009 Jun [cited 2015 Oct 28];86(3):466–71. Available from: https://www.sciencedirect.com/science/article/pii/S0034528808002051?via%3Dihub 10.1016/j.rvsc.2008.08.009 18947842

[43] Fagbemi BO, Obarisiagbon IO. Comparative evaluation of the enzyme-linked immunosorbent assay (ELISA) in the diagnosis of natural *Fasciola gigantica* infection in cattle. Vet Q [Internet]. 1990 1 [cited 2015 Dec 19];12(1):35–8. Available from: http://www.tandfonline.com/doi/abs/10.1080/01652176.1990.9694239 10.1080/01652176.1990.9694239 2321350

[44] Rehman T, Khan MN, Abbas RZ, Babar W, Sikandar A, Zaman MA. Serological and coprological analyses for the diagnosis of *Fasciola gigantica* infections in bovine hosts from Sargodha, Pakistan. J Helminthol [Internet]. 2015 24 [cited 2015 Dec 17];1–9. Available from: http://journals.cambridge.org/abstract_S0022149X15000711 10.1017/S0022149X15000711 26300295

[45] Krishna Murthy CM, Souza PED. An enzyme-linked immunosorbent assay for diagnosis of *Fasciola gigantica* infection in cattle and buffaloes. J Parasit Dis [Internet]. 2015 [cited 2016 Jul 2];39(4):783–5. Available from: https://www.ncbi.nlm.nih.gov/pmc/articles/PMC4675568/pdf/12639_2013_Article_414.pdf. 10.1007/s12639-013-0414-2 PMC4675568 26688653

[46] Piedrafita D, Raadsma HW, Prowse R, Spithill TW. Immunology of the host–parasite relationship in fasciolosis ( Fasciola hepatica and *Fasciola gigantica* ). Can J Zool [Internet]. 2004 15 [cited 2015 Oct 28];82(2):233–50. Available from: http://www.nrcresearchpress.com/doi/abs/10.1139/z03-216#.XCYPiVX7SUk

[47] Raadsma HW, Kingsford NM (2008). Suharyanta, Spithill TW, Piedrafita D. host responses during experimental infection with Fasciola gigantica and Fasciola hepatica in merino sheep: II. Development of a predictive index for Fasciola gigantica worm burden. Vet Parasitol.

[48] Mazeri S, Sargison N, Kelly RF, Bronsvoort BM de C, Handel I. Evaluation of the performance of five diagnostic tests for *Fasciola hepatica* infection in naturally infected cattle using a Bayesian no gold standard approach. PLoS One. 2016;11(8). 10.1371/journal.pone.0161621 PMC5001639 27564546

[49] Ortiz PL, Claxton JR, Clarkson MJ, McGarry J, Williams DJ. The specificity of antibody responses in cattle naturally exposed to *Fasciola hepatica*. Vet Parasitol [Internet]. 2000 10;93(2):121–34. Available from: https://www.sciencedirect.com/science/article/pii/S0304401700003605?via%3Dihub. 10.1016/s0304-4017(00)00360-5 11035230

[50] Wamae LW, Hammond JA, Harrison LJ, Onyango-Abuje JA. Comparison of production losses caused by chronic *Fasciola gigantica* infection in yearling Friesian and Boran cattle. Trop Anim Health Prod [Internet]. 1998 [cited 2015 Nov 12];30(1):23–30. Available from: https://link.springer.com/article/10.1023/A:1005057225427. 10.1023/a:1005057225427 9719825

[51] Abebe R, Abunna F, Berhane M, Mekuria S, Megersa B, Regassa A. Fasciolosis: Prevalence, financial losses due to liver condemnation and evaluation of a simple sedimentation diagnostic technique in cattle slaughtered at Hawassa Municipal abattoir, southern Ethiopia [Internet]. Vol. 14, Ethiopian Veterinary Journal. Ethiopian Veterinary Association; 2010 [cited 2015 Jul 21]. p. 39–52. Available from: https://www.ajol.info/index.php/evj/article/view/63868

[52] Mingoas KJP, Zoli PA, Tchoumboue J, Ebene NJ, Toukala JP (2014). Socio-economic characteristics and husbandry practices of cattle breeders in the Vina division. Cameroon Int J Livest Prod.

[53] Hammond IA, Sewell MMH. Diseases caused by helminths. In: Sewell MMH, Brocklesby DW, editors. Handbook on animal diseases in the tropics. 4th Editio. London: Bailliere Tindall; 1990. p. 105–160.

[54] Awah-Ndukum J, Tchoumboue J, Niba AT. Prevalence of bovine tuberculosis at the SODEPA Douala abattoir, Cameroon (1995–2003). Cameroon J Exp Biol [Internet]. 2006 31 [cited 2015 Jul 17];1(2):116–20. Available from: https://link.springer.com/article/10.1007/s11250-005-5688-7

[55] Keyyu JD, Monrad J, Kyvsgaard NC, Kassuku AA. Epidemiology of Fasciola gigantica and amphistomes in cattle on traditional, small-scale dairy and large-scale dairy farms in the southern highlands of Tanzania. Trop Anim Health Prod [Internet]. 2005 May;37(4):303–14. Available from: http://www.ncbi.nlm.nih.gov/pubmed/15934638. 10.1007/s11250-005-5688-7 15934638

[56] Spithiil TW, Smooker PM, Copeman DB, Dalton JP (1999). *Fasciola gigantica*: epidemiology, control immunology and molecular biology. Fasciolosis. First Edit.

[57] World Bank. Intensification of livestock production systems in the north west region of Cameroon: a south-to-south collaboration for technology transfer, the Tugi Silvopastoral project [internet]. Washington DC; 2012. Available from: http://documents.worldbank.org/curated/en/2012/01/16496156/intensification-livestock-production-systems-north-west-region-cameroon-south-to-south-collaboration-technology-transfer-tugi-silvopastoral-project

[58] Egbe NF, Muwonge A, Ndip L, Kelly RF, Sander M, Tanya V, et al. Abattoir-based estimates of mycobacterial infections in Cameroon. Nat Sci Reports. 2016;6(24320). 10.1038/srep24320. 10.1038/srep24320 PMC4830956 27075056

[59] R Core Team. R: A Language and Environment for Statistical Computing [Internet]. Vienna, Austria; 2014. Available from: http://www.r-project.org/.

[60] Wickham H. ggplot2: elegant graphics for data analysis [Internet]. Springer New York; 2009. Available from: http://had.co.nz/ggplot2/book

[61] Fernandez-i-Marin X (2016). Analysis of MCMC samples and Bayesian inference. J Stat Softw.

[62] Robin X, Turck N, Hainard A, Tiberti N, Lisacek F, Sanchez JC (2011). pROC: an open-source package for R and S+ to analyse and compare ROC curves. BMC Bioinformatics.

[63] Hui SL, Walter SD. Estimating the Error Rates of Diagnostic Tests. Biometrics [Internet]. 1980 Mar [cited 2018 Jun 7];36(1):167. Available from: https://www.jstor.org/stable/2530508?origin=crossref 7370371

