# Supplementary figures and images for: Assessing the performance of a Fasciola gigantica serum antibody ELISA to estimate prevalence in cattle in Cameroon
Source: BMC Vet Res. 2019 Jan 3;15:8. doi: 10.1186/s12917-018-1762-z (PMC6318879; doi:10.1186/s12917-018-1762-z)

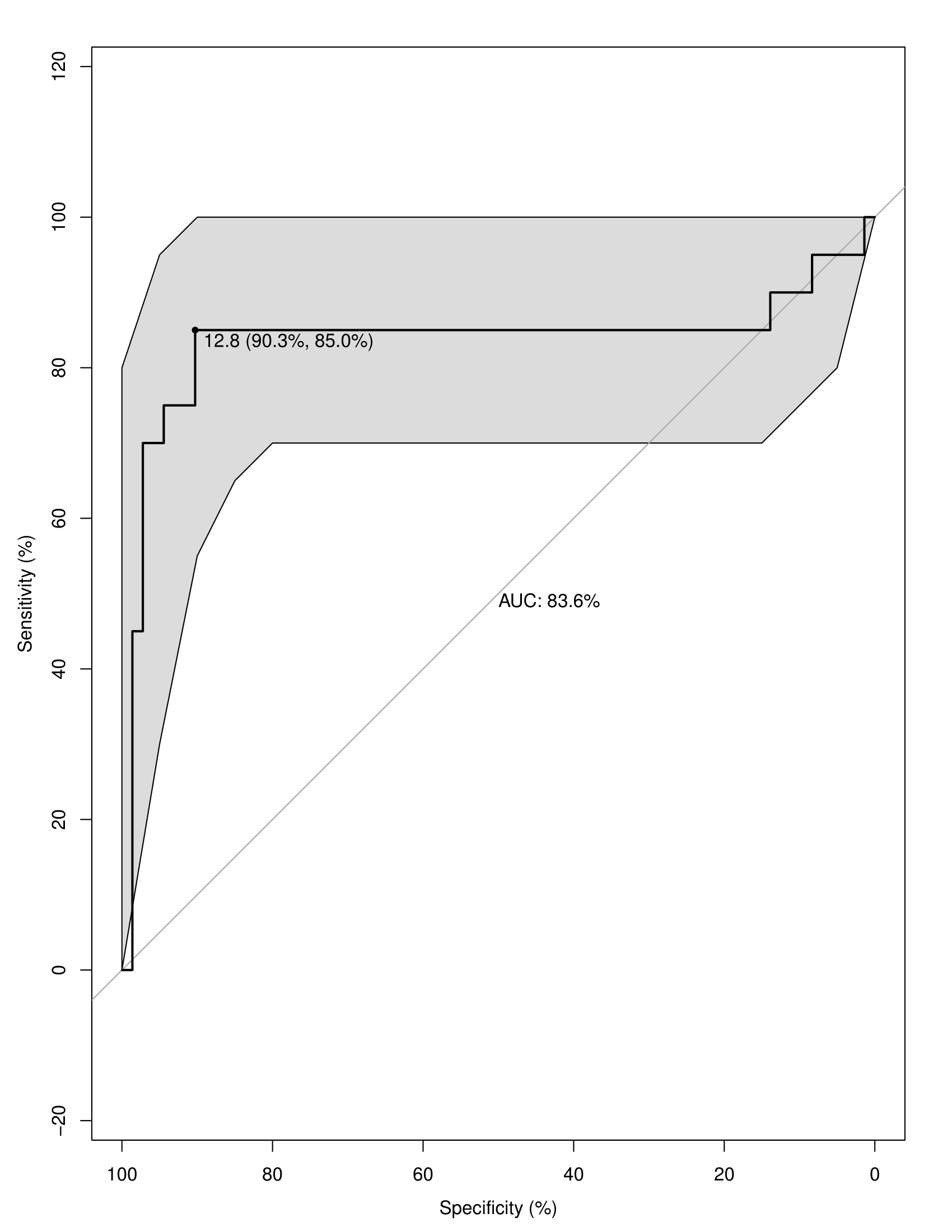

Supplement: Supplementary file 1 — The receiver operator curve (ROC) for the F. gigantica antibody ELISA of F. gigantica positive (n = 20) and negative (n = 72) cattle. The SE and SP of the ELISA is represented on the y and x axes respectively. The line (“Staircase trace”) represents SE and SP, with 95% CI being the grey shaded area, at different positive cut-off values for the ELISA. A selected positive cut-off value of 12.8 PP balances sensitivity (85.0%) and specificity (90.3%) with an AUC of 83.6%. (TIFF 42 kb) [file 12917_2018_1762_MOESM1_ESM.tiff]
